# *In Vivo* Blockade of Murine ARTC2.2 During Cell Preparation Preserves the Vitality and Function of Liver Tissue-Resident Memory T Cells

**DOI:** 10.3389/fimmu.2018.01580

**Published:** 2018-07-09

**Authors:** Björn Rissiek, Marco Lukowiak, Friederike Raczkowski, Tim Magnus, Hans-Willi Mittrücker, Friedrich Koch-Nolte

**Affiliations:** ^1^Department of Neurology, University Medical Center, Hamburg-Eppendorf, Hamburg, Germany; ^2^Institute of Immunology, University Medical Center, Hamburg-Eppendorf, Hamburg, Germany

**Keywords:** ADP-ribosylation, P2X7, tissue-resident memory T cells, nanobodies, ARTC2.2

## Abstract

On murine T cells, GPI-anchored ADP-ribosyltransferase 2.2 (ARTC2.2) ADP-ribosylates the P2X7 ion channel at arginine 125 in response to nicotinamide adenine dinucleotide (NAD^+^) released during cell preparation. We have previously shown that chronic gating of P2X7 by ADP-ribosylation reduces the vitality and function of regulatory T cells and natural killer T cells that co-express high levels of ARTC2.2 and P2X7. Here, we evaluated the expression of ARTC2.2 and P2X7 by effector and memory T cells in the liver of naïve mice and after infection with *Listeria monocytogenes* (Lm). We found that KLRG1^−^/CD69^+^ tissue-resident memory T cells (Trm) in the liver of naïve mice and 7 weeks after infection with Lm express high levels of ARTC2.2 and P2X7. Isolation of liver Trm and subsequent incubation at 37°C resulted in cell death of the majority of CD4^+^ and CD8^+^ Trm. Injection of the ARTC2.2-blocking nanobody s+16a 30 min prior to organ harvesting effectively prevented ADP-ribosylation of P2X7 during cell preparation and thereby prevented NAD-induced cell death of the isolated Trm upon subsequent incubation at 37°C. Consequently, preserving Trm vitality by s+16a injection enabled a highly sensitive *in vitro* cytokine expression profile analyses of FACS sorted liver Trm. We conclude that *in vivo* blockade of ARTC2.2 during cell preparation by nanobody s+16a injection represents a valuable strategy to study the role and function of liver Trm in mice.

## Introduction

Mammalian ecto-ADP-ribosyltransferases (ecto-ARTs) are a family of toxin-related enzymes that use extracellular (NAD^+^) to attach an ADP-ribose group to arginine residues of cell surface proteins. In mice, the ecto-ARTs family comprises six family members (ARTC1–5) including two isoforms of ARTC2, termed ARTC2.1 and ARTC2.2 that are encoded by two closely linked genes (*Art2a* and *Art2b)* (1). ARTC2 isoforms are expressed on immune cells. While ARTC2.1 is expressed mainly by innate immune cells such as macrophages, dendritic cells, and microglia, ARTC2.2 is the major ecto-ART expressed by T cells (2–4). The ARTC2 enzymes ADP-ribosylate various target proteins and thereby modulate their function. One well-characterized target of ARTC2.2-mediated ADP-ribosylation is the adenosine triphosphate (ATP)-gated P2X7 ion channel (5, 6). Two differentially spliced isoforms of P2X7 are expressed by murine immune cells (7, 8). P2X7a is expressed by innate immune cells and plays a critical role in inflammasome formation and the release of mature interleukin (IL)-1β from these cells. P2X7k is expressed by T cells where ADP-ribosylation of P2X7 at R125 can trigger gating of P2X7k at much lower concentrations of NAD^+^ compared to ATP (9). ATP and ADP-ribosylation-mediated gating of P2X7 on T cells induces the rapid influx of calcium, activation of cell surface metalloproteases, cleavage of cell surface ecto-domains of CD62L (10) and CD27 (11), externalization of phosphatidylserine, and ultimately cell death (5).

Several studies have shown that the ecto-ART substrate NAD^+^ can be released from endogenous sources, e.g., *via* cell lysis or, in a more controlled fashion, *via* connexin hemichannels (12, 13). We have previously demonstrated that NAD^+^ is released during the passage of cell culture cells and the preparation of primary leukocytes from murine spleen, lymph nodes, or the liver (12, 14). Of note, ARTC2 is catalytically active and ADP-ribosylates cell surface proteins, including P2X7, even if cells are prepared at 4°C (12). Gating of P2X7 by ADP-ribosylation, however, requires temperatures above 24°C, i.e., functional effects of P2X7 ADP-ribosylation on T cells are manifested during reincubation of isolated T cells at 37°C. This commonly results in cell death of a substantial fraction of T cells (12), in particular T cell populations that co-express high levels of ARTC2.2 and P2X7 such as regulatory T cells (Tregs) and natural killer T cells (NKTs) (14, 15). ADP-ribosylation of P2X7 during cell preparation affects the vitality of these cells and makes it difficult to use them for further *in vitro* functional assay or for adoptive transfer experiments (16). We recently described an experimental approach to prevent preparation-related ADP-ribosylation by systemic injection of the ARTC2.2-blocking nanobody s+16a, a 15 kDa small single domain antibody derived from llama heavy chain antibodies (14, 17). Injection of s+16a 30 min prior to sacrificing the mice prevents the detrimental effects of preparation-related P2X7 ADP-ribosylation and facilitates the use of freshly prepared Tregs and NKTs for functional assay and adoptive transfer experiments.

Tissue-resident memory T cells (Trm) comprise a population of T cells, which stays in peripheral tissues after an immune response against invading pathogens, forming a rapid first-line defense against recurring infection (18). Trm are characterized by cell surface expression of CD69 and lack of cell surface expression of the killer cell lectin-like receptor subfamily G member 1 (KLRG1) (19). A recent study suggests that cell preparation affects the vitality and function of this T cell population in the context of a malaria mouse model (20). In our present study, we analyzed liver Trm from naïve mice and from mice 7 weeks after *Listeria monocytogenes* (Lm) infection in order to increase the number of Trm in the liver. In both, we analyzed the expression of ARTC2.2 and P2X7. We tested the impact of the ARTC2.2-blocking nanobody s+16a on the vitality of Trm vitality and on the functional capacity of freshly prepared Trm to secrete cytokines. Our results demonstrate that CD8^+^ and CD4^+^ liver Trm co-express high levels of ARTC2.2 and P2X7 and that preparation of primary Trm from liver causes ADP-ribosylation of P2X7 resulting in cell death in the majority of isolated CD4^+^ and CD8^+^ Trm upon incubation at 37°C. Systemic injection of nanobody s+16a preserved Trm vitality and allowed sensitive monitoring of otherwise unnoticed cytokine expression.

## Materials and Methods

### Mice

C57BL/6 mice were used for all experiments. ARTC2ko mice (21) and P2X7 mice (22) were backcrossed onto the C57BL/6J background for at least 12 generations. Splenocytes from RAG1ko mice (23) were used as feeder cells in some experiments. All mice were bred at the animal facility of the University Medical Center (UKE). All experiments involving tissue derived from animals were performed with approval of the responsible regulatory committee (Hamburger Behörde für Gesundheit und Verbraucherschutz, Veterinärwesen/Lebensmittelsicherheit, G17/17). All methods were performed in accordance with the relevant guidelines and regulations.

### Lm Infection

C57BL/6 mice were intravenously (i.v.) infected with a Lm strain recombinant for ovalbumin (2 × 10^4^ bacteria in 200 µl PBS) (24). Mice were housed under specific pathogen-free conditions in individually ventilated cages, received food and water *ad libitum* and were controlled on a daily basis during the experiment.

### Nanobody s+16a Treatment

s+16a was recombinantly produced by transfecting HEK-6E cells with the pCSE2.5 vector containing the coding region of s+16a. Mice were injected i.v. with 50 µg of the ARTC2.2-blocking nanobody s+16a solved in 100 µl NaCl 30 min prior to sacrificing the mice in order to prevent ADP-ribosylation of P2X7 during cell preparation.

### Preparation of Liver Trm

Mice were anesthetized by CO_2_/O_2_ exposure and sacrificed by cervical dislocation. The preparation of single-cell suspensions from liver was performed throughout at 4°C. Liver lobes were gently mashed through a metal sieve using a syringe piston. Purification of liver leukocytes was achieved by running a Percoll gradient. For this, cells were resuspended in 5 ml 33% Percoll/PBS in a 15-ml Falcon tube, and centrifuged at 1,600 rpm, 12°C, for 20 min without breaks. The pellet was collected, and cells were washed once in PBS (ThermoFisher). Contaminating erythrocytes were lysed using ACK erythrocyte lysis buffer (155 mM NH_4_Cl, 10 mM KHCO_3_, 0.1 mM EDTA, pH 7.2). For FACS analyses or sorting, cells were washed and resuspended in FACS buffer containing PBS, 1 mM EDTA (Sigma), and 0.1% bovine serum albumin (Sigma).

### Antibodies and Flow Cytometry

The following antibodies were used for flow cytometric analyses: anti-ARTC2.2 (clone Nika109; UKE), anti-CD3 (clone 145-2C11, BioLegend), anti-CD4 (clone RM4–5, BioLegend), anti-CD8 (clone 53-6.7, BioLegend), anti-CD45 (clone30-F11, BioLegend), anti-CD69 (clone H1.2F3, BioLegend), anti-KLRG1 (clone 2F1/KLRG1, BioLegend), and anti-P2X7 (clone RH23A44, UKE). PE-labeled CD1d-tetramer (PBS-57-loaded) was kindly provided by the NIH tetramer core facility. Flow cytometric analyses were performed on a BD Fortessa (Beckton Dickinson) or a BD FACS CantoII (Beckton Dickinson). Liver Trm were identified as CD4^+^CD69^+^KLRG1^−^ or CD8^+^CD69^+^KLRG1^−^, and tissue residency was probed by applying the anti-CD45 *in vivo* labeling technique (25). For this, 2 µg of fluorochrome-labeled anti-CD45-perCP solved in 100 µl PBS were intravenously injected into mice, which were sacrificed 3 min after injection. After cell preparation, all leukocytes were labeled with anti-CD45-PE-Cy7 and blood vessel resident cells were identified as CD45-PE-Cy7^+^CD45-perCP^+^. For some experiments, liver Trm were sorted at the FACS Core Facility at the University Medical Center Hamburg-Eppendorf (UKE) on a BD FACSAriaFusion (Beckton Dickinson). Analysis of flow cytometric data was performed using FlowJo X (Flowjo, LLC).

### Monitoring P2X7-Induced Cell Death

CD4^+^CD69^+^KLRG1^−^, CD4^+^CD69^−^KLRG1^+^, CD8^+^CD69^+^KLRG1^−^, and CD8^+^CD69^−^KLRG1^+^ cells were FACS sorted and 1 × 10^4^ cells were resuspended in 200 µl complete IMDM medium containing IMDM (ThermoFisher) + 5% FCS, β-mercaptoethanol (50 µM, ThermoFisher), and gentamicin (50 µg/ml, ThermoFisher). For some experiments, FACS sorted Trm were cultured in the presence of 2 × 10^5^ eFluor^670^-labeled feeder cells obtained from RAG1ko mice in a ration of 1:20. Cells were incubated for 2 h at 4°C on ice or at 37°C in a cell culture incubator in the presence of propidium iodide (PI, 2.5 µg/ml, ImmunoChemistry Technologies, LLC). PI uptake was used to determine cell death by flow cytometry directly after incubation.

### Cytokine Secretion Assay

CD4^+^CD69^+^KLRG1^−^ and CD8^+^CD69^+^KLRG1^−^ Trm were isolated by FACS sorting from mice 7 weeks after infection with Lm. Isolated Trm were cultured in 200 µl IMDM (ThermoFisher) + 5% FCS, β-mercaptoethanol (50 µM, ThermoFisher), and gentamicin (50 µg/ml, ThermoFisher) at a cell density of 20,000 (CD4^+^ Trm) or 10,000 (CD8^+^ Trm) cells per well for 20 h in the presence of Phorbol 12-myristate 13-acetate (PMA, 20 ng/ml, Invivogen) and ionomycin (1 µg/ml, Invivogen) to induce cytokine expression. Levels of IFN-γ, TNF-α, IL-2, IL-4, IL-21, IL-22, IL-17A, IL-17F, IL-10, IL-9, IL-5, and IL-13 were measured in the supernatants of stimulated Trm by using the LEGENDplex mouse Th cytokine 13-plex (BioLegend) according to the manufacture’s instruction.

## Results

### Liver Trm Co-Express ARTC2.2 and P2X7

**Figure 1:**
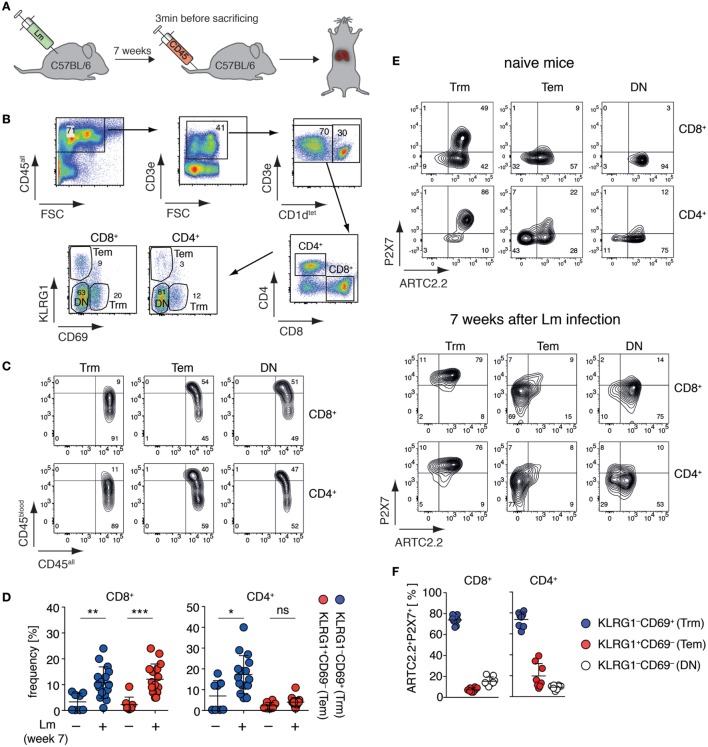
Liver Trm co-express high levels of ARTC2.2 and P2X7. **(A)** C57BL/6 mice were infected i.v. with 2 × 10^4^ *Listeria monocytogenes* (Lm). Seven weeks after infection, mice were treated with perCP-labeled anti-CD45 3 min before sacrificing to label vascular leukocytes. The liver of treated mice was harvested for Trm analyses. **(B)** Gating strategy: within the CD3^+^CD1d^tet−^ T cell pool Trm were identified as CD8^+^CD69^+^KLRG1^−^ or CD4^+^CD69^+^KLRG1^−^ and effector memory T cells (Tem) as CD8^+^CD69^−^KLRG1^+^ or CD4^+^CD69^−^KLRG1^+^; double negative (DN) marks CD8^+^ or CD4^+^ T cells that were CD69^−^KLRG1^−^. **(C)** *In vivo* anti-CD45 labeling (CD45^blood^) of Trm, Tem, and DN in relation to *ex vivo* anti-CD45 labeling (CD45^all^). **(D)** Frequencies of CD8^+^ and CD4^+^ Trm and Tem in the liver of naïve and Lm infected mice. Two groups were compared using Student’s *t*-test (*n* = 8–16) with *p < 0.05, **p < 0.01, ***p < 0.001. **(E)** FACS analyses of ARTC2.2 and P2X7 expression on Trm, Tem, and DN cells from naïve mice (upper panel) and mice 7 weeks after infection with Lm (lower panel). **(F)** Frequency of ARTC2.2 and P2X7 co-expressing cells from mice 7 weeks after Lm infection are quantified as % of CD4^+^ or CD8^+^ T cells. The shown data represent results from at least two independently performed experiments.

Tissue-resident memory T cells (Trm) are a population of non-circulating CD4^+^ and CD8^+^ T cells that stay in peripheral tissues after infection to build a rapid first-line defense against recurring pathogen invasion (18). A recent study suggests that liver CD8^+^ Trm are affected by NAD^+^ released during cell preparation (20), however, these cells have not yet been fully characterized toward their expression of ARTC2.2 and P2X7. Since co-expression of ARTC2.2 and P2X7 potentially renders cells susceptible toward NAD^+^-induced cell death (5), we set out to measure ARTC2.2 and P2X7 expression on these cells. In order to increase the frequency of liver Trm, we infected mice i.v. with Lm and analyzed liver CD4^+^ and CD8^+^ Trm 7 weeks after infection (Figure 1A). CD4^+^ and CD8^+^ Trm were identified as CD3^+^CD1d^tet−^ T cells that express CD69 but lack KLRG1 expression. Conversely, CD69^−^KLRG1^+^ T cells were identified as effector memory T cells (Tem). The remaining CD69^−^KLRG1^−^ T cells were termed “double negative” (DN) including naïve and memory T cells (Figure 1B). In order to distinguish vascular T cells and from tissue-resident T cells, we injected anti-CD45-perCP antibodies 3 min prior to sacrificing the mice. Due to the fenestrated endothelium of the liver sinusoids, anti-CD45-perCP *in vivo* labeling (termed CD45^blood^) led to a low-level CD45 staining of all CD45^+^ liver leukocytes (termed CD45^all^). However, when comparing CD8^+^ and CD4^+^ Trm with Tem or DN T cells, only Tem and DN T cells contained a substantial fraction of cells that were strongly labeled by the i.v. injected anti-CD45-perCP antibody, confirming that CD69^+^KLRG1^−^ Trm reside deeper in the liver tissue (Figure 1C). Seven weeks after Lm infection, the frequencies of CD8^+^ Trm and Tem as well as of CD4^+^ Trm were significantly increased compared to naïve mice (Figure 1D). We next, analyzed these three subpopulations of CD8^+^ and CD4^+^ T cell populations obtained from naïve mice and 7 weeks after Lm infection for expression of ARTC2.2 and P2X7 using specific monoclonal antibodies (26, 27). In naïve mice, a substantial fraction of CD8^+^ and CD4^+^ Trm co-express high levels of ARTC2.2 and P2X7 (Figure 1E). By contrast, most CD8^+^ and CD4^+^ Tem and DN cells express ARTC2.2 but lack P2X7 expression. Seven weeks after Lm infection, we found that the majority of CD8^+^ and CD4^+^ Trm co-express high levels of ARTC2.2 and P2X7. By contrast, most CD8^+^ and CD4^+^ Tem express only low levels of ARTC2.2 and P2X7. Furthermore, DN CD8^+^ and CD4^+^ T cells do not express substantial levels of P2X7 but a major fraction of CD8^+^ (>80%) and CD4^+^ (>60%) DN T cells expresses high levels of ARTC2.2. In summary, CD8^+^ and CD4^+^ liver Trm co-express high levels of ARTC2.2 and P2X7, especially when isolated 7 weeks after Lm infection (Figure 1F), and therefore are potentially sensitive toward NAD^+^ released during cell preparation.

### Injection of s+16a Preserves the Vitality of Isolated Liver Trm

**Figure 2:**
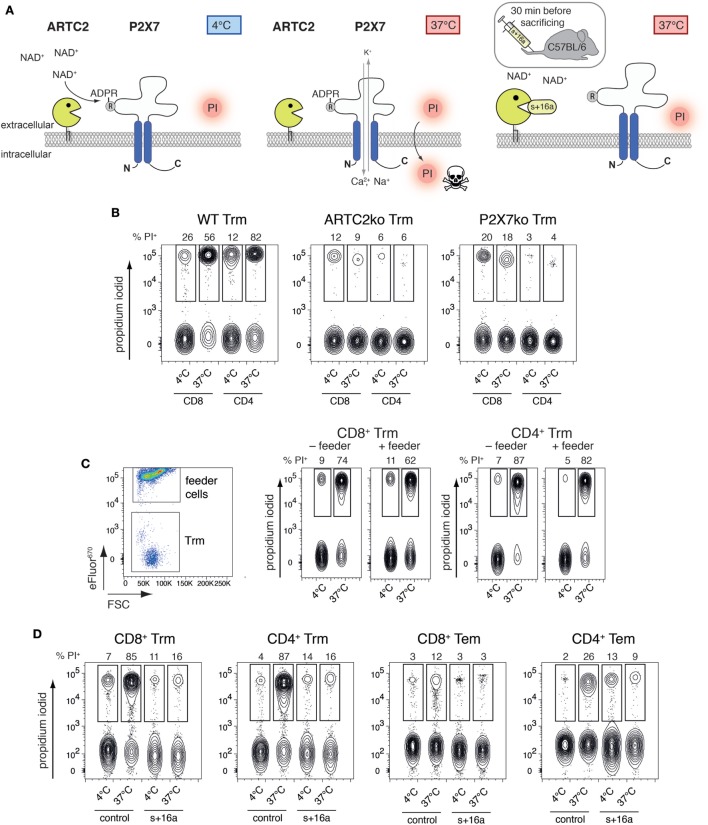
ADP-ribosylation of Trm during cell preparation induces cell death upon 37°C incubation. **(A)** NAD^+^ is released during cell preparation and serves as substrate for ARTC2.2 to ADP-ribosylate P2X7 at R125, even if cells are prepared at 4°C. ADP-ribosylation-mediated gating of P2X7 occurs when cells are brought back to 37°C, inducing Ca^2+^ influx and ultimately cell death. ADP-ribosylation of P2X7 during cell preparation and subsequent activation of P2X7 at 37°C can be prevented by injection of the ARTC2.2-blocking nanobody s+16a 30 min prior to sacrificing the mice. **(B)** CD8^+^ and CD4^+^ Trm were isolated *via* FACS from the liver of naïve WT, ARTC2ko, and P2X7ko mice. Cells were incubated at 37°C for 2 h and propidium iodide (PI) uptake was measured by flow cytometry as marker for cell death. **(C)** Isolated CD8^+^ and CD4^+^ Trm from the liver of naïve WT mice were cultured in the presence or absence of eFluor^670^-labeled feeder cells in a ration of 1:20. Cells were incubated at 37°C for 2 h and PI uptake by Trm was measured by flow cytometry as marker for cell death. **(D)** CD8^+^ and CD4^+^ Trm and effector memory T cells (Tem) were isolated *via* FACS from the liver of Lm infected mice 7 weeks after infection. One group of mice was treated with s+16a prior to organ harvesting and the second group was left untreated as control. Cells were incubated at 37°C for 2 h and PI uptake was measured by flow cytometry as marker for cell death. The shown data represent results from two independently performed experiments.

T cells co-expressing high levels of ARTC2.2 and P2X7, such as Treg and NKT cells, are highly susceptible to NAD^+^-released during cell preparation, resulting in reduced vitality and function of the isolated cells (14, 16). Extracellular NAD^+^ released during cell preparation serves as substrate for ARTC2.2 catalyzing the ADP-ribosylation of R125 of P2X7, even when cells are prepared at 4°C (Figure 2A). When cells are brought back to 37°C, e.g., for functional assays or adoptive transfer, ADP-ribosylation of P2X7 triggers channel gating leading to influx of Ca^2+^ and ultimately to cell death which can be visualized by PI uptake. ADP-ribosylation of P2X7 during cell preparation can be prevented by injection of the ARTC2.2-blocking nanobody s+16a 30 min prior to organ harvesting. Since liver Trm co-express high levels of ARTC2.2 and P2X7, we hypothesized that preparation-related ADP-ribosylation of P2X7 reduces the vitality of these cells upon reincubation at 37°C. To test this, we first isolated Trm from the liver of naïve WT, ARTC2ko, and P2X7ko mice *via* FACS and incubated the isolated cells for 2 h in IMDM + 5% FCS in the presence of PI at 37°C or kept the cells at 4°C on ice. PI uptake by Trm was subsequently analyzed by flow cytometry as a measure for cell death. We observed that the majority of WT CD8^+^ and CD4^+^ Trm died upon incubation at 37°C as shown by incorporation of PI. This discrepancy in vitality upon incubation at 4 and 37°C was virtually absent when analyzing CD8^+^ and CD4^+^ Trm from ARTC2ko and P2X7ko mice (Figure 2B). To further test whether the presence of feeder cells could improve the vitality of Trm, we co-incubated FACS-sorted WT CD8^+^ and CD4^+^ Trm with eFluor^670^-labeled splenocytes obtained from RAG1ko mice (Figure 2C). The results show that the presence of feeder cells does not improve the vitality of CD8^+^ and CD4^+^ liver Trm, when incubated at 37°C. Together, this suggests that the observed loss of Trm vitality upon incubation at 37°C is triggered *via* ARTC2.2-mediated ADP-ribosylation of P2X7. In order to further probe this conclusion, we compared the vitality of isolated CD8^+^ and CD4^+^ Trm and Tem obtained from mice 7 weeks after Lm infection and analyzed the impact of injecting the ARTC2.2-blocking nanobody s+16a 30 min before sacrifice on cell vitality. For this, one group of mice was injected i.v. with 50 µg s+16a 30 min prior to sacrifice, the other group was left untreated as control. As shown before for liver Trm from naïve mice, we observed that the vast majority (85%) of CD8^+^ and CD4^+^ Trm of the untreated control group died during the 37°C incubation, compared to only 12% of CD8^+^ Tem and 26% of CD4^+^ Tem (Figure 2D). By contrast, CD8^+^ and CD4^+^ Trm and Tem sorted from the s+16a-treated mice exhibited a preserved vitality with only 16% dead cells after incubation at 37°C. Together, our results reveal the detrimental effects of preparation-related P2X7 ADP-ribosylation on liver Trm vitality upon incubation at 37°C.

### Injection of s+16a Allows Cytokine Profiling of Liver Trm

**Figure 3:**
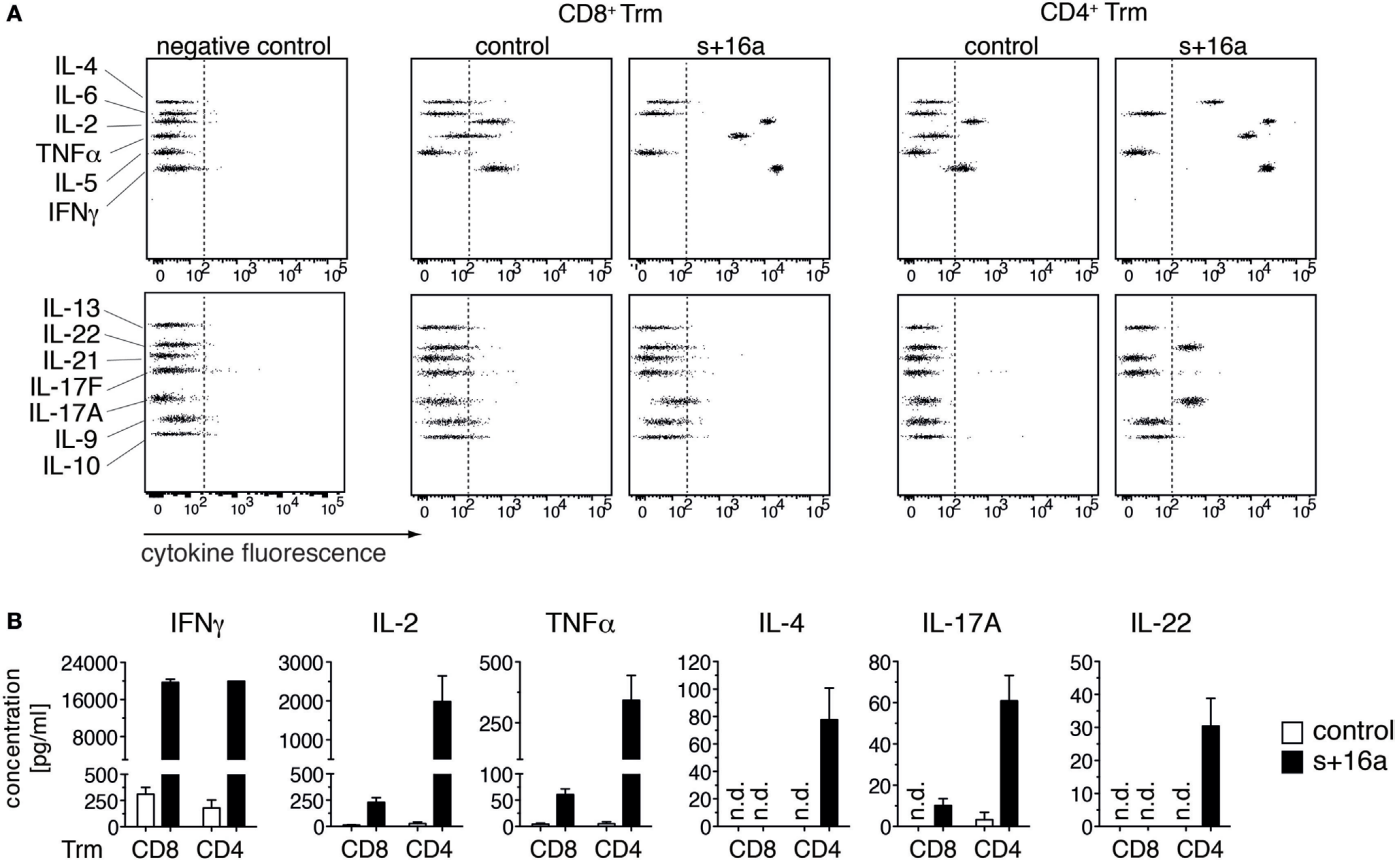
*In vitro* cytokine expression profile of PMA/ionomycin stimulated liver Trm. **(A)** CD8^+^ (1 × 10^4^ cells) and CD4^+^ (2 × 10^4^ cells) Trm from the liver of Lm infected mice were FACS sorted 7 weeks after infection. One group of mice was treated with s+16a prior to organ harvesting and the second group was left untreated as control. Cells were stimulated with PMA/ionomycin for 20 h and T cell cytokines [IFNγ, TNFα, interleukin (IL)-2, IL-4, IL-21, IL-22, IL-17A, IL-17F, IL-10, IL-9, IL-5, and IL-13] were measured in the supernatants using a bead-based 13-plex assay. **(B)** The concentration of cytokines in the supernatants of stimulated CD4^+^ and CD8^+^ Trm from s+16a-treated and control mice (*n* = 3) was quantified.

Our findings that the majority of isolated liver Trm succumb to cell death upon incubation at 37°C raises the question whether this affects functional assays that involve incubation steps at 37°C and impinges on the quality of the obtained data. To test this, we analyzed the cytokine expression profile of freshly sorted liver CD8^+^ and CD4^+^ Trm from s+16a-treated mice and untreated control mice 7 weeks after infection with Lm in a proof-of-principle experiment. We restimulated 1–2 × 10^4^ isolated cells *in vitro* with PMA/ionomycin for 20 h and analyzed the cytokine expression profile in the cell supernatants using a 13-plex bead-based immunoassay designed to quantify T cell-specific cytokine responses. By this, we detected low concentrations of IFN-γ, TNF-α, and IL-2 in the supernatants of stimulated CD8^+^ Trm and CD4^+^ Trm isolated from control mice (Figure 3A). Strikingly, CD8^+^ and CD4^+^ Trm isolated from s+16a-treated mice produced more than 100-fold higher concentrations of IFN-γ, TNF-α, and IL-2 (Figure 3B). Furthermore, no IL-4 or IL-22 and only very low levels of IL-17A were detectable in the supernatants of stimulated CD4^+^ Trm isolated from control mice. By contrast, CD4^+^ Trm harvested from s+16a-treated mice showed robust expression of IL-4, IL-22, and IL-17 upon PMA/ionomycin stimulation (Figures 3A,B). Interestingly, IL-17A was also detectable in the supernatants of stimulated CD8^+^ Trm from s+16a-treated mice. These results demonstrate that ADP-ribosylation of P2X7 during cell preparation reduces the vitality of Trm and therefore blunts their cytokine secretion during 37°C PMA/ionomycin stimulation. This can limit the detection of low-level expressed cytokines, as demonstrated for IL-17A, IL-22, and IL-4.

## Discussion

Our results show that liver tissue-resident memory T cells (Trm) co-expresses high levels of ARTC2.2 and P2X7. The high ARTC2.2 expression enables ADP-ribosylation of Trm cell surface proteins during cell preparation from the liver in response to NAD^+^ released during cell preparation. ARTC2.2 ADP-ribosylates the P2X7 ion channel even if cells are prepared at 4°C. Subsequent incubation of the isolated Trm at 37°C then induces P2X7 activation, resulting in cell death and making it difficult to use these cells during further *in vitro* assays. The consequences of preparation-related ADP-ribosylation on Trm resemble those of ARTC2.2 and P2X7 co-expressing Tregs and NKT cells, i.e., dramatic loss of cell vitality during *in vitro* culture (14). Our rescue approach, i.e., injection of the ARTC2.2-blocking nanobody s+16a prior to sacrificing the mice, markedly preserves the vitality of Trm at 37°C incubation and allows sensitive *in vitro* cytokine expression profiling.

Several studies have shown that both, ARTC2.2 and P2X7 are differentially expressed among T cell subpopulations (15, 28–31). Downregulation of ARTC2.2 is observed by T cells upon T cell receptor activation (32). Similarly, recently activated T cells express lower levels of P2X7 compared to their naive counterparts (33). Both findings fit to the phenotype of liver CD69^−^KLRG1^+^ Tem observed in the Lm infected mice. The physiological role of ARTC2.2 and P2X7 on Trm remain unclear. High P2X7 expression on Trm could be beneficial to boost T cell receptor signaling toward eliciting a T cell memory response during pathogen reencountering since P2X7 can act as receptor for autocrine ATP stimulation to enhance IL-2 production (34). However, massive tissue damage during liver infection accompanied by the release NAD^+^ would probably kill most of the liver Trm and thereby delay pathogen clearance. Therefore, further studies are needed to investigate the interplay between ARTC2.2 and P2X7 on Trm *in vivo* during a second course of infection. Furthermore, it needs to be investigated whether Trm from other organs also exhibit a high co-expression of ARTC2.2 and P2X7 and are vulnerable to NAD-induced cell death (NICD). A recent study by Yoshizawa et al. describes the transcriptome characterization of CD69^+^ Trm in the lung of influenza infected mice. Interestingly, the authors found a differential P2X7 expression in two Trm populations directed against the two immunodominant epitopes PA_224–233_/H-2D^b^ and NP_366–374_/H-2D^b^. PA_224–233_/H-2D^b^ Trm exhibited pronounced P2X7 mRNA expression in CD103^−^ and, at slightly lower level, in CD103^+^ cells. By contrast, P2X7 mRNA expression was virtually absent in CD103^−^ and CD103^+^ NP_366–374_/H-2D^b^ Trm (35). Another study demonstrated that CD4^+^ T cells from the small intestine of naïve mice exhibit P2X7 expression on CD69^+^ cells (36). The latter were susceptible toward NICD as demonstrated by *in vivo* depletion after injection of NAD^+^, indicating that ARTC2.2 is co-expressed by these cells.

In general, it is advised to check the expression levels of ARTC2.2 and P2X7 when working with murine T cell populations. As a first approach, this can be done by querying public mRNA sequencing databases such as www.immgen.org (37). The results of such analyses reveal that Tregs, CD4^+^ memory T cells, and NKT cells from spleen and liver express high mRNA levels of *Art2* and *P2rx7*. These cells can then be characterized for ARTC2.2 and P2X7 cell surface expression by flow cytometry or by functional assays that monitor P2X7-related effects such as ecto-domain shedding of CD27 and CD62L or phosphatidylserine externalization and PI uptake upon 37°C incubation (16). It is important to note that the human ARTC2 gene is non-functional due to premature stop codons and therefore ARTC2-related effects observed in mice are not directly transferrable to humans (38).

ARTC2.2 ADP-ribosylates multiple targets on T cells, including CD25, the α-chain of the high affinity IL-2 receptor (39). Here, ADP-ribosylation reduces binding of IL-2 and subsequent STAT5 signaling in Tregs. For CD8^+^ cytotoxic T cells, it has been shown that ARTC2.2-catalyzed ADP-ribosylation of CD8 diminishes the binding to MHC class I (40). Therefore, the beneficial effects of systemically injected ARTC2.2 blocking nanobody on Trm vitality and cytokine secretion may be mediated also in part by preventing ADP-ribosylation of these and other targets. A recently published study describes a mass-spectrometry-based approach to identify ADP-ribosylated proteins (41). This technique has been applied by our group to identify ADP-ribosylated cell surface target proteins on microglia (4) including several cell adhesion molecules. This technique could be utilized to analyze the ADP-ribosylome of T cells in order to identify other target proteins that are potentially influenced in their function by ADP-ribosylation.

One limitation of our approach to prevent ADP-ribosylation during T cell preparation using the s+16a nanobody is that it needs to be injected i.v. or i.p. into mice which may be technically challenging and requires the approval to perform animal experiments. A recent study suggests to use the P2X7 antagonist KN62 as alternative substance that can be used *in vitro* during the preparation of single-cell suspensions from lymphoid organs in order to protect T follicular helper and regulatory cells from NICD and further during *in vitro* culture of T cells (42). Indeed, this preserved the vitality of ARTC2.2 and P2X7 expressing T follicular helper and regulatory cells. However, P2X7 blockade by KN62 does not prevent the ADP-ribosylation of other membrane proteins during cell preparation. Moreover, blockade of P2X7 might also interfere with T cell function, as autocrine ATP stimulation upon T cell receptor activation enhances the production of IL-2 *via* P2X7 activation (34). Furthermore, though KN62 is a highly potent non-competitive P2X7 antagonist (IC50: 15 nM), it is also cell permeable and a selective inhibitor for Ca^2+^/calmodulin-dependent protein kinase II (IC50: 500 nM), which plays a role in T cell receptor mediated IkB kinase activation (43). Therefore, even though systemic injection of the s+16a nanobody approach is technically more elaborative, blocking of ARTC2.2 during cell preparation probably interferes less with T cell function compared to P2X7 blockade.

In a proof-of-principle experiment, we demonstrated that injection of s+16a allows a sensitive *ex vivo* cytokine expression analyses of isolated Trm in response to polyclonal PMA/ionomycin stimulation. By this, we could detect low-level cytokines expressed by CD4^+^ Trm such as IL-4, IL-17A, and IL-22 that were undetectable when CD4^+^ Trm were isolated from control mice that did not receive s+16a treatment. It is likely that the reduced vitality of the isolated control CD4^+^ Trm is responsible for this, however, we cannot rule out that tissue-resident T-helper type 1 (Th1), 2 (Th2), 17 (Th17), and 22 (Th22) cells are differentially affected by NICD. Indeed, P2X7 is highly expressed by intestinal Th1 and Th17 cells and injection of NAD^+^ induces the depletion of these cells as it did for intestinal Tregs (36).

We conclude that when working with murine liver Trm, one needs to be aware that T cells expressing high levels of ARTC2.2 and P2X7 succumb to preparation-related ADP-ribosylation of P2X7 and other cell surface proteins that affects Trm vitality and function. Using our nanobody-based approach to block ARTC2.2 *in vivo* minimizes cell loss, paving the way for sensitive Trm cytokine expression profiling and other functional analyses.

## Ethics Statement

This study was carried out in accordance with the German animal welfare law. The protocol was approved by the Hamburger Behörde für Gesundheit und Verbraucherschutz, Veterinärwesen/Lebensmittelsicherheit (approval number G17/17).

## Author Contributions

BR, ML, and FR performed the experiments and analyzed the data. TM, H-WM, and FK-N supervised the experiments and assisted with data interpretation and manuscript preparation. BR assembled the figures and wrote the manuscript, which has been reviewed by all authors.

## Conflict of Interest Statement

FK-N receives royalties from sales of antibodies developed in the lab *via* MediGate GmbH, a 100% subsidiary of the University Medical Center, Hamburg. All other authors declare that the research was conducted in the absence of any commercial or financial relationships that could be construed as a potential conflict of interest. Nanobody s+16a can be obtained *via* material transfer agreement from FK-N.
